# Pedestrian Inattention Blindness While Playing Pokémon Go as an Emerging Health-Risk Behavior: A Case Report

**DOI:** 10.2196/jmir.6596

**Published:** 2017-04-01

**Authors:** Stefania Barbieri, Gianna Vettore, Vincenzo Pietrantonio, Rossella Snenghi, Alberto Tredese, Mauro Bergamini, Sara Previato, Armando Stefanati, Rosa Maria Gaudio, Paolo Feltracco

**Affiliations:** ^1^ Department of Urgent and Emergency Care University of Padova Padova Italy; ^2^ Preventive Medicine and Risk Assessment University of Ferrara Ferrara Italy; ^3^ Forensic Medicine and Toxicology University of Ferrara Ferrara Italy; ^4^ Department of Legal Medicine University of Padova Padova Italy

**Keywords:** Pokémon Go, videogames, pedestrians, multiple trauma, traffic accidents, motor vehicle collisions, road injuries

## Abstract

**Background:**

Cases of trauma resulting from the use of mobile phones while driving motor vehicles have become quite common in recent years. Road injuries incurred by people playing video games on mobile phones (or other media devices) while walking have also become a cause for concern. Pokémon Go has been the world's most popular game since it was launched in July 2016, with more than 15 million players trying to catch all Pokémon available in the game; however, the case detailed here is the first reported accident in the medical literature caused by a pedestrian distracted by the game while crossing a street.

**Objective:**

We aim to provide additional information on the innovative nature of distractions that generate risks in road-users, and to explore the underreporting of pedestrian-motor vehicle collisions due to mobile device usage.

**Methods:**

We included in this case report a 25-year-old male who suddenly crossed a road while playing Pokémon Go and was hit by a van, reporting several injuries and being assisted by the Emergency Medical Service of our hospital (Padova, Italy). The patient’s history, the circumstances in which the collision happened, imaging data, and clinical course information were recorded per our hospital’s privacy policy.

**Results:**

The patient hit by the van was playing Pokémon Go on his mobile phone while crossing a street, despite red traffic lights, which he did not notice due to of the distraction induced by the game.

**Conclusions:**

Mobile videogames that imply movement (ie, walking, running, cycling) to play are an effective way to improve physical activity practice, especially in adolescents and young adults. Nevertheless, cases like the one presented here point out that these games could pose a significant risk to users who play while walking, cycling, or driving in unsafe areas such as city streets, because players become distracted and may ignore surrounding hazards. Comprehensive, multilevel interventions are needed to reduce accidents caused by distraction, and to stress findings on the positive and negative effects of video games, which are becoming a source of public health concern. Health care providers should be aware of their chief role in these possible prevention strategies, based on their direct interactions with road incident victims.

## Introduction

Cases of trauma, and even death, resulting from the use of mobile phones while driving motor vehicles have become quite common in recent years. Road injuries incurred by people playing video games on mobile phones (or other media devices) while walking have also become a cause for concern [1-5]. Several authors have reported the combination of distracted walking or driving as a frequent cause of road traffic incidents; mobile phones are a well-known cause of distraction [3]. However, only a very small number of injured individuals confess to have been using the mobile phone while walking or driving. Despite widespread and tighter regulations covering the use of mobile phones while driving, mobile phone distraction rates remain high in young drivers, and often result in fatal incidents [3,5,6]. The use of mobile phones (and other media devices) is not regulated for pedestrians, and considering virtual reality games are particularly popular among young people, emergency departments will probably see a rise in trauma cases caused by the use of video games while walking. Nasar and Troyer showed that pedestrians, like drivers, reported injuries resulting from distraction due to the use of mobile phones or digital devices [3]. In a study promoted by the Department of Health Promotion, Bungum et al [7] described the behavior of 866 individuals: approximately 20% of walkers were distracted as they crossed streets because they were talking on a mobile phone, eating, drinking, smoking, or they were walking inside the crosswalk while a red light was flashing against them. These circumstances were critical in many of the incidents reported [7]. As reported by several authors, mobile phone-related injuries among pedestrians lead to increased morbidity due to distracted attention and unsafe behavior during their use, and users risk serious injury, falls, collisions with obstacles, or even death [8,9].

Trauma in emergency departments may present health care providers with a myriad of unforeseen clinical scenarios, and the anamnestic data entered must reflect the modern context. Pokémon Go and other virtual-reality applications for mobile phones and media devices meant to be used while walking or biking have the advantage of providing entertainment in outdoor activities for children and young people, thus favoring physical exercise [3,7,8,10]. However, the indiscriminate use of mobile phones and other virtual-reality games can also pose a health hazard, as pedestrians, bikers, and drivers distracted by the games have a higher risk of causing motor vehicle collisions, or becoming their victims. More than 270,000 pedestrians die on the world’s roads each year, accounting for 22% of all road traffic deaths (1.24 million total) [11]. In addition, in 2013 more than 150,000 pedestrians were treated in emergency departments for nonfatal crash-related injuries [12]. Some reports describe situations in which an accident occurred when car drivers or pedestrians were using hand-held mobile phones. In 2014, Basch et al [1] observed walking behaviors in New York pedestrians and found that one in four pedestrians (>3500 individuals observed) were distracted by mobile electronic devices while crossing roads during the *walk* (28.8%) and *do not walk* (26.3%) signals.

**Figure 1:**
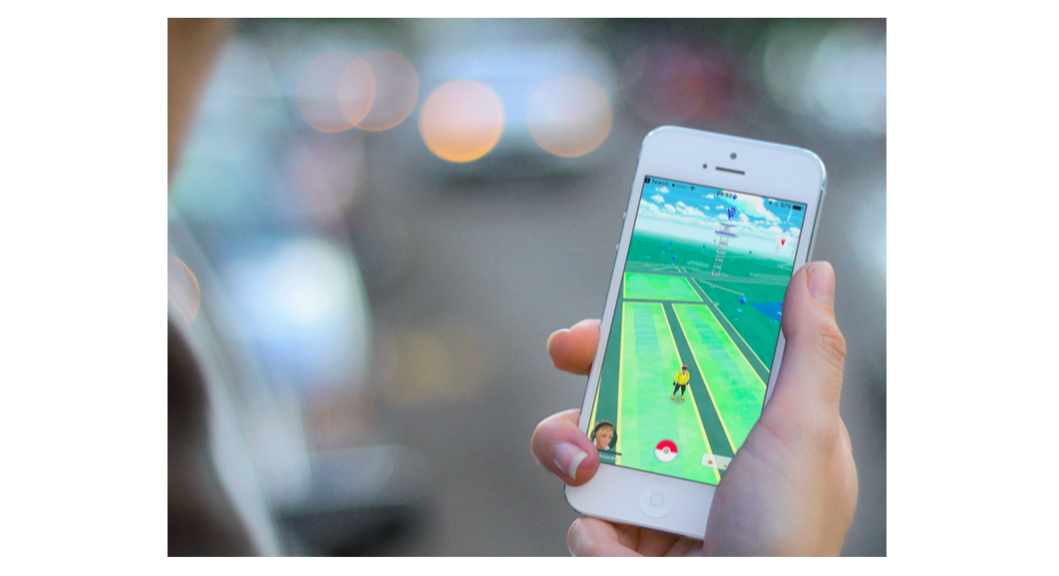
Pokémon Go is a virtual reality smartphone-based videogame.

Pokémon Go has been the world's most popular game since it was launched in July 2016, with more than 15 million players trying to catch all Pokémon available in the game; however, the case reported here is the first accident described in the medical literature caused by a pedestrian distracted by the game while crossing a street. In a recent car collision in Vienna, the driver stopped in the middle of the street while playing Pokémon Go. When questioned by a police officer, the driver admitted that he and his passengers were playing the game [13]. Pokémon Go may become particularly dangerous if the player attempts to gain levels and *Gym Prestige* by completing the *Pokedex*, which involves capturing the rarest Pokémon and requires the most effort. This goal is potentially the most distracting activity in the game, although it does represent a strong motivation to increase walking speed and distance (Figure 1).

Here we briefly report details of a serious motor vehicle accident involving a young pedestrian, who was distracted while playing Pokémon Go as he was crossing a residential road with a traffic light signaling against him. The purpose of this paper is to provide additional information on the innovative nature of distractions that generate risks in road-users, and to explore the underreporting of pedestrian-motor vehicle collisions due to mobile device usage.

After obtaining appropriate patient consent, a thorough review of the incident scene, the patient's hospital records (prehospital and emergency room sources), and official police-reported incident data were examined.

## Methods

**Figure 2:**
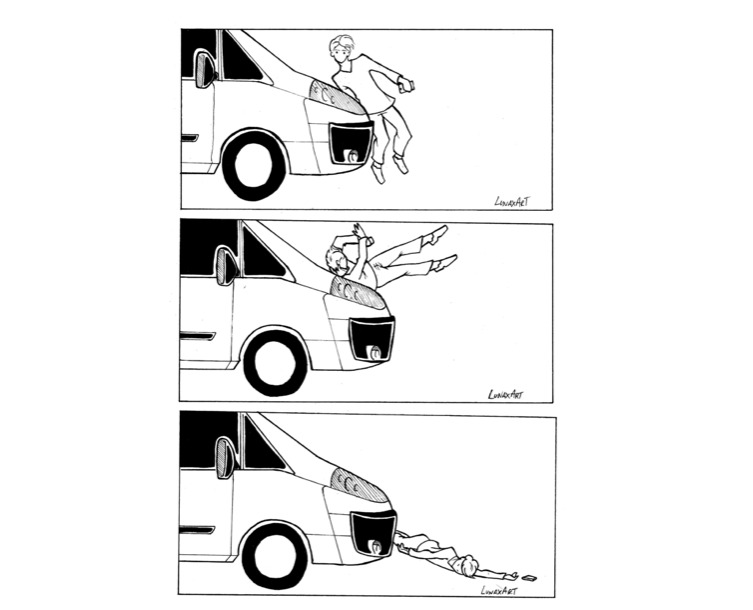
The pedestrian-vehicle collision sequence.

This paper describes a case report a 25-year-old male who suddenly crossed a road while playing Pokémon Go and was hit by a van (Figure 2), reporting several injuries and being assisted by the Emergency Medical Service of our hospital (Padova, Italy). The patient’s history, the circumstances in which the collision happened, imaging data, and clinical course information were recorded per our hospital’s privacy policy.

## Results

**Figure 3:**
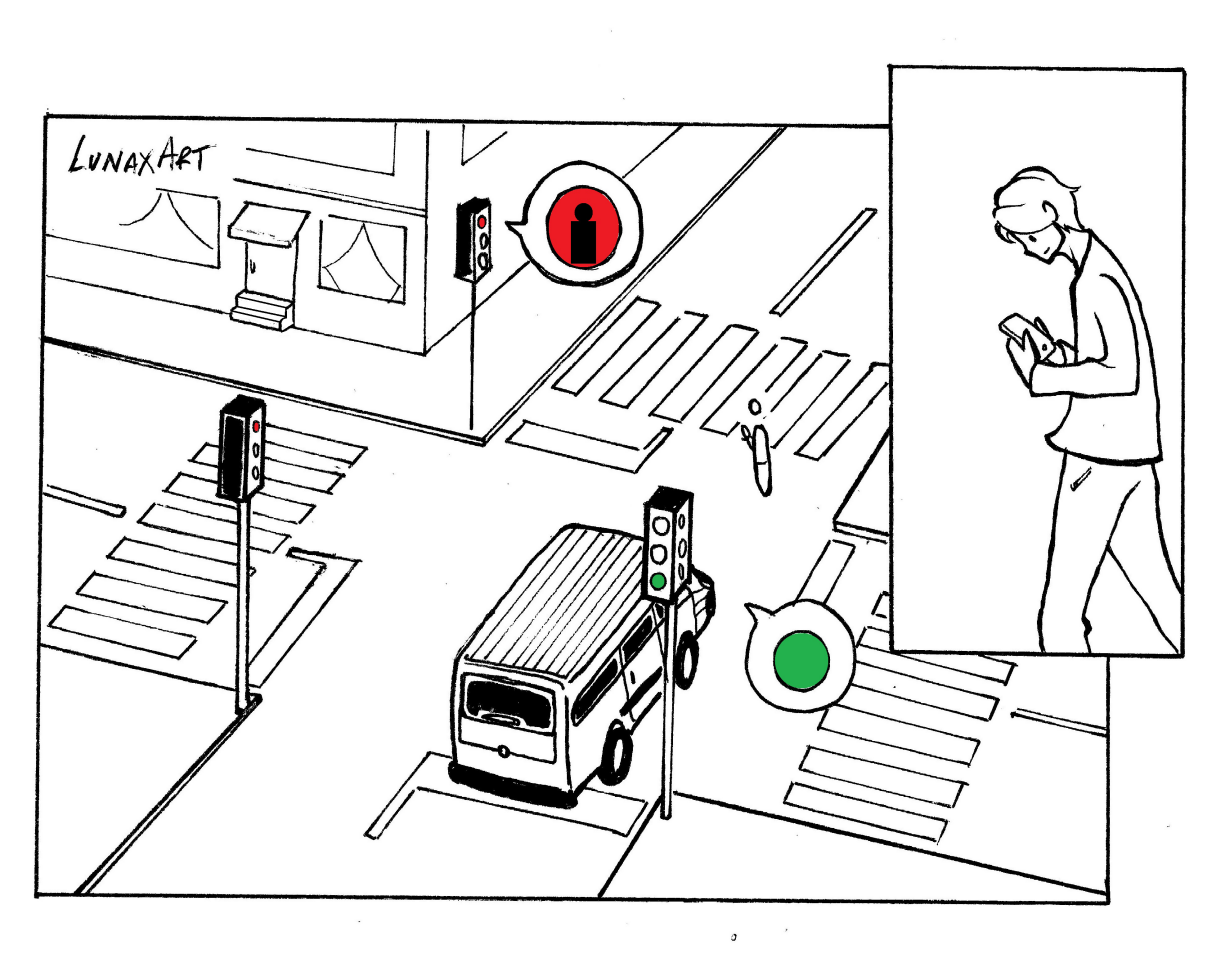
Circumstances and setting of the incident.

The patient had been walking fast, on his own, on the sidewalk of a residential street when he unexpectedly, “jumped into the road at the intersection without attempting to look in any direction,” as described by the van driver; witnesses to the incident provided similar accounts (Figure 3). The patient collided with the front of a 3-ton van driven by a man who did not expect the event. According to the driver’s expectation of the young man’s movements immediately before the collision, the driver could not have foreseen his intention, as the youth’s eyes were focused on his smartphone. The speed of the van was compatible with legal restrictions and with the conditions of the street, and the driver avoided a more severe impact by slamming on his brakes.

Later analysis of the incident showed that the young man’s violation of the red light had caused the accident, that the van was travelling at a reasonable speed, and that the violence of impact was compatible with a short man-vehicle distance (high-energy trauma). According to the young man’s statement, he was distracted; he was intensely involved in the game, and neither looked at the traffic light nor heard the noise of a rapidly approaching vehicle.

According to the existing literature, pedestrian simulation models are based on flow dynamics, traffic conditions, vehicle type, road geometry, characteristics of pedestrian behavior, and police reports. Table 1 lists the details as a report covering some aspects of the context of this particular road incident, and its potential contributing factors.

**Table 1:** Characteristics of the collision.

Parameter	Details
**Pedestrian**	
	Male
	25 years old
	No alcohol or drugs
	University student
	Wore glasses
	Pedestrian travel speed (calculated 4.6 kilometers/hour)
	Pedestrian kinematics followed a typical *wrap* trajectory
	Injury type: lower and upper extremities, thorax
	Distraction due to playing Pokémon Go: no perception of crossing device
**Environment**	
	12:00 AM; high-volume traffic
	Residential area (speed limit 50 kilometers/hour)
	Pedestrian crosswalk
	Red traffic light for pedestrian
**Vehicle**	
	Van
	Number of vehicles involved in a collision=1
**Driver**	
	Male
	50 years old
	No alcohol or drug abuse
**Dynamic**	
	Vehicle speed <40 kilometers/hour
	Distance pedestrian-van: 10 meters
	Lateral collision
	Contact point pedestrian-van: (1) primary injuries, and (2) secondary injuries
	Injured body regions: lateral collision
	Pedestrian impact kinematics: the young man was thrown onto the van bonnet and projected towards the windscreen; he landed on the van roof, and then fell to the ground

**Figure 4:**
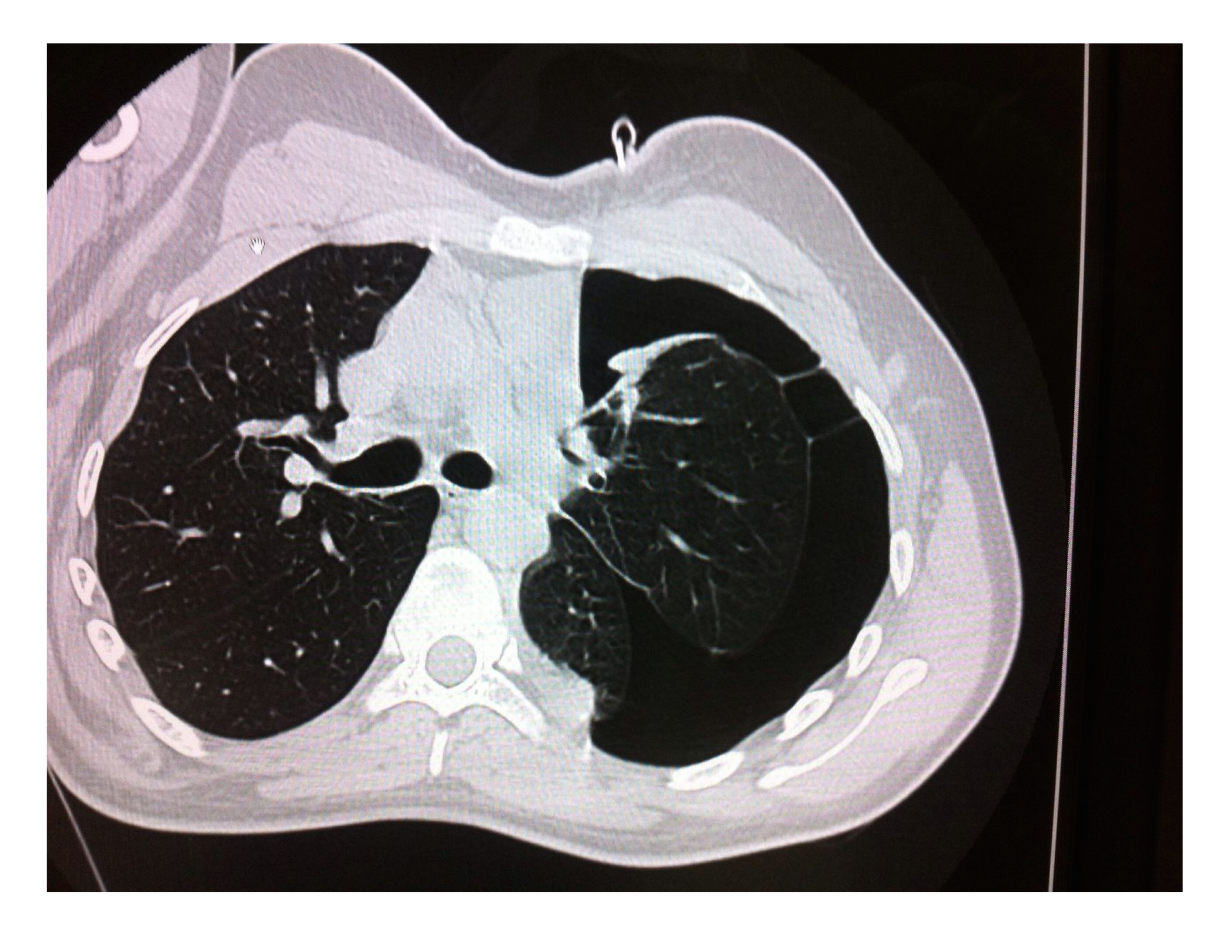
Left-sided pneumothorax due to the collision.

The severity of the young man’s injuries was related to many factors, including vehicle speed and the angle at which the impact occurred. The patient sustained blunt closed thoracic and lower limb trauma. In the prehospital setting the patient complained of intense pain in many areas (posterior head and neck, right arm, right leg, abdomen) but was hemodynamically stable and almost completely conscious. The patient was transferred directly from the scene of the accident to the hospital and immediately subjected to computerized tomography scanning, which revealed multiple high-energy traumas due to major injuries. In accordance with *Advanced Trauma Life Support* guidelines, a complete primary survey was carried out, including diagnostic chest X-rays and pelvic X-rays. The initial *Focused Assessment with Sonography for Trauma* examination was followed by a secondary survey with a whole-body computerized tomography scan. The powerful impact of the van had caused multiple fractures to the ribs, and a left-sided pneumothorax was identified (Figure 4), together with fractures of the left femur and right tibia. No brain damage was detected. A pleural drainage system was inserted, and the lower limb fractures were stabilized. In-hospital stay was lengthy (10 days), but no major respiratory problems or infectious diseases occurred, and repair to the lower limb fractures was uneventful.

## Discussion

Walking and physical activity are important factors in reducing noncommunicable diseases, including cardiovascular disease, cancers, obesity, type 2 diabetes, and impaired quality of life. Health professionals have noted the potential benefits of Pokémon Go smartphone games, which have reached millions of people and improved outdoor physical activity and social interactions. To increase the chances of catching Pokémon characters and Pokémon *eggs*, participants are encouraged to walk through city streets as an extra activity, viewing the world through their mobile phones and using their cameras for 2, 5, or 10 kilometers (while incubating an egg for a new character to be born) [10].

The purpose of this paper is to provide additional and specific information on the underreporting of pedestrian-motor vehicle collisions. Knowledge of the sequence of events during the impact of the distracted pedestrian and the vehicle has been crucial for proper reconstruction of the incident, along with vehicle speed at the moment of the impact, how far the person was thrown after impact, and when the driver braked. The responsibilities of emergency medicine physicians consists of: (1) preventing severe morbidity due to injury patterns manifested in pedestrian trauma; (2) describing the incident scene and reconstructing pedestrian-vehicle collisions; and (3) identifying the mechanism(s) of injury, the initial and final positions of the pedestrian, and the sequence of events leading to trauma.

In the accident described here, the dynamic response of the pedestrian resulted in the characteristic injuries of pedestrian-road vehicle collisions in terms of severity of injury, body posture, direction of force, impact injuries and subsequent contact with the road surface, direction of acting force, and when exactly the incident happened. The interview was conducted to analyze and report data to determine all causes of the incident which, in this particular case, was due to playing a videogame in hazardous conditions. Although many studies and prevention programs have been implemented to curtail pedestrian injuries, this case is important for a better understanding of the reasons why this accident occurred, and how to plan for future preventive actions.

This case report confirms the high risk of injury to pedestrians using mobile phones, especially if playing Pokémon Go, which is a very new form of distraction. This is the first case study concerning a road incident with injuries to a pedestrian in which the essential cause was *Pokémon Go game search*. Such cases are helpful for pediatricians and physicians to recognize this phenomenon and its risks, and proper knowledge is important for the prevention and early detection of adolescents’ at-risk behaviors. The risks associated with distracted walking, biking, or driving can be analyzed from several viewpoints when considering the relationship between distraction and inattention as a prevalent causative factor of road incidents.

### Conclusions

Mobile apps that integrate game play with physical exercise, such as Pokémon Go, lead to substantial physical activity increases and reach sedentary populations [10]. In the case presented here, the need to capture virtual Pokémon creatures in real locations represents a strong motivation to increase one’s walking distance and speed, but safety while walking must still be considered. The positive and negative consequences of gaming indicate that, although facilitating improved daily physical activity, gaming may result in low vigilance and secondary task distraction (a well-known phenomenon that contributes to road accidents), especially in young people.

Available data clearly indicate that distraction while moving around city streets could potentially represent a serious public health problem. Interventions should be implemented following research-based recommendations, and improved knowledge of the context is an important consideration (in this case, behaviors concerning road traffic safety). The case presented here emphasizes the difficulties involved in identifying the circumstances of a pedestrian trauma and shows the importance of implementing common strategies to reduce road users’ risks. Comprehensive, multilevel interventions and further research projects are needed to reduce incidents caused by distraction, and to more thoroughly study the positive and negative effects of video games.

Health care providers should be aware of their chief role in these possible prevention strategies, based on their direct interactions with road incidents victims. Pokémon Go was released very recently, and no follow-up studies have examined the long-term effectiveness of encouraging players to increase physical activity levels while following Pokémon creatures. Therefore, we still do not know whether the benefits of playing the game outweigh the risks, and further research over a longer observation period is needed.
